# A compact triple wideband mimo antenna for microwave, ku, and mm-wave band applications of 5g wireless communication

**DOI:** 10.1371/journal.pone.0347785

**Published:** 2026-06-01

**Authors:** Abdulhameed N. Hameed, Samir Salem Al-Bawri, Md. Mushfiqur Rahman, Samia Larguech, Md. Mhedi Hasan, Mohamad A. Alawad, Md. Shabiul Islam, Mohammad Tariqul Islam

**Affiliations:** 1 Department of Electrical, Electronic and Systems Engineering, Faculty of Engineering and Built Environment, Universiti Kebangsaan Malaysia (UKM), Bangi, Selangor, Malaysia; 2 Space Science Centre, Climate Change Institute, Universiti Kebangsaan Malaysia (UKM), Bangi, Selangor, Malaysia; 3 Centre Centre for Advanced Devices and Systems, CoE of Robotics and Sensing Technologies, Multimedia University, Cyberjaya, Selangor, Malaysia; 4 Department of Electrical Engineering, College of Engineering, Princess Nourah bint Abdulrahman University, Riyadh, Saudi Arabia; 5 Department of Information and Communication Technology (ICT), Faculty of Engineering, Comilla University, Cumilla, Bangladesh; 6 Faculty of Artificial Intelligence and Engineering, Multimedia University, Cyberjaya, Selangor, Malaysia; 7 Department of Electrical Engineering, College of Engineering, Imam Mohammad Ibn Saud Islamic University (IMSIU), Riyadh, Saudia Arabia; Parul University, INDIA

## Abstract

This work introduces a compact triple wideband Multiple-Input Multiple-Output (MIMO) antenna specifically designed for 5G applications. The antenna was designed simply by integrating three structures, each optimally nominated to operate within a specific frequency band. Subsequently, the antenna’s bandwidth was enhanced by incorporating a slot on the front side and an L-shaped structure on the rear side. The compact dimension of the antenna is about 37.5 × 37.5 × 1.6 mm^3^, corresponding to an electrical size of 0.375λ × 0.375λ × 0.016λ at 3 GHz. The antenna is fabricated and measured. Measurement results reveal that the developed antenna shows a fractional bandwidth of about 90.91% (5 GHz), 39.9% (6.6 GHz) and 20.4% (5 GHz) (Simulated: 101.7(40.6 GHz)) in the Sub-6 gigahertz, Ku and mm-Wave bands, respectively for |S11| < - 10dB. The proposed antenna achieves measured peak gains of 5 dBi and simulated gains of 5.5 dBi, and 10.1 dBi for the Sub-6, Ku, and mm-Wave bands, respectively for |S11| < -10dB. It has excellent diversity performance, with envelope correlation coefficients (ECC) of less than 0.06, 0.002 and 0.002 for Sub-6, Ku and mm-Wave bands respectively and diversity gains (DG) greater than 9.68, 9.99 and 9.99 for the Sub-6, Ku and mm-Wave bands respectively. Good TARC values are observed at 90-degree phase for all bands. In addition, the accepted simulated CCL values are observed for all bands. Also, the simulated and the measured MEG lies within −3 dB to −4.3 dB for all bands. Moreover, the radiation patterns in the H-plane of all bands are like omnidirectional patterns and in the E plane the antenna exhibits monopole like radiation patterns for the Sub 6 and Ku bands. The versatile multiband operation makes the designed antenna a reliable solution for advanced wireless communication systems.

## 1 Introduction

In recent years, significant advancements in wireless communication technologies have enabled the seamless integration of fifth generation (5G) technology. This integration has facilitated the interconnection of billions of devices within a unified wireless network [1]. These connected devices operate across various frequency bands to support diverse applications. For this reason, multiband antennas found their potential in various applications namely nano satellite, global positioning systems, 5G mm Wave applications and narrowband IoT [2–5]. The (1–6) GHz band is reserved for sub-6 GHz band of 5G which covers bands for mobile broadband, wireless backhaul, WiFi and other applications of wireless communications [6,7]. The (7–8) GHz band is allocated for satellite communications and other services [8]. The (12–18) GHz range, known as the Ku band, is employed for satellite communications and radar systems [9]. The (24–100) GHz range includes frequencies allocated for 5G millimeter-wave (mm-Wave) communications, enabling ultra-high-speed data transfer and low-latency communication for advanced applications [10]. To meet the stringent requirements of modern 5G applications, antenna systems need to be operated efficiently across sub-6 GHz, Ku, and mm-Wave frequency ranges [11]. This necessity has led to extensive research efforts to develop multiband antennas capable of seamless performance across all these bands, ensuring reliable and high-speed wireless communication [12,13]. Integrating these bands into a single antenna system presents several challenges. One of the primary difficulties is achieving a compact multiband antenna system that can support the multiple frequency ranges. The significant difference in wavelengths between these bands necessitates antenna systems capable of operating efficiently at both low and high frequencies while maintaining a small physical layout. Most existing designs either support a single frequency band or require large physical structures to accommodate these ranges.

Sub-6 GHz and Ku band frequencies are advantageous for long-distance communication, offering lower attenuation and broader coverage, albeit at the expense of data rates [14]. Conversely, the mm-wave spectrum enables significantly higher data rates but is constrained to short-range transmission due to increased path loss and atmospheric absorption [15–21]. Higher transmission loss at higher frequencies counters an antenna’s enhanced gain and efficiency, requiring an improved received signal. [22]. Designing a multiband antenna within the same structure introduces additional complexities, including optimizing antenna size, achieving high data rates, ensuring sufficient isolation between frequency bands, and maintaining adequate antenna gain. These challenges highlight the intricate balance required to develop efficient and compact antennas for seamless operation over the sub-6 GHz and mm-Wave 5G bands. These issues might be resolved by combining the advantages of millimeter-wave and microwave frequencies into a high-performance incorporated MIMO antenna [23].

MIMO technology enables multiple data streams to transmit and receive various data streams simultaneously using multiple antennas at both transmitter and receiver ends. The technological method ensures enhanced spectral efficiency alongside increased data rates to support the demanding communication systems that the rapidly developing 5G network requires [24]. The development of compact wideband MIMO antennas for 5G applications has received a lot of interest in recent times [25]. Several modern studies have focused on Sub-6 GHz MIMO antennas to support 5G coverage in the lower frequency spectrum. For instance, a compact 4- element Sub-6 GHz flexible monopole antenna was proposed for 5G wireless communications, utilizing two-arm structure and coplanar waveguide. The design achieved compactness and flexibility but did not integrate higher-frequency bands critical for 5G’s high data rate requirements [26]. In the branch of mm-wave MIMO antennas, a printed compact MIMO antenna configuration and good diversity characteristics was developed for 5G mm-Wave applications, offering ultra- wideband operation spanning from 25 GHz to 50 GHz by employing Defective Ground Structure (DGS) technique. While effective for mm-wave applications, this design did not support Sub-6 GHz frequencies, limiting its suitability for integrated multiband operation [27]. Recent efforts have also explored the Ku band for satellite and backhaul applications. DGS technique is also utilized to design compact MIMO antennas. A miniaturized MIMO antenna for C, X, and Ku band applications was introduced, featuring a dual-element design with a DGS and a tapered microstrip feed line. While suitable for satellite communication, the design did not address the need for multiband integration, particularly with mm-Wave and Sub-6 GHz spectrums [28]. A multibranch antenna with U-shaped DGS is proposed to compact and multiband radiation characteristics where the DGS section is tuned to achieve good impedance matching the low frequency resonance [29]. Rifaqat Hussain et el. proposed a transmission line and power divider-based radiator with four concentric pentagonal DGS slots to get compact and multiband radiation characteristics. The largest pentagonal slot causes the low frequency resonance [30]. In another research, an integration of circular ring and square shaped ring is used as the main radiating patch where a U-shaped DGS is used to get low frequency resonance [31]. Sidra Jabeen and Qasim Umar Khan proposed an eight element MIMO antenna where the sub 6 GHz band is excited by two L-shaped monopoles and one annular ring antenna. In this work, a large rectangular slot is cut on the ground plane which assists the radiator to form a compact antenna [32]. These literatures though achieved compact size with DGS structures but still are not compact enough compared to another research work like [33]. In [33], a dual sided three-stage stair is backed by a partial ground plane where the partial ground plane causes a band in the flower frequency region. Despite these advancements, integrating Sub-6 GHz, Ku, and mm-Wave bands into a single compact MIMO antenna remains largely unexplored. Existing designs often focus on specific frequency ranges, sacrificing compactness, gain, or bandwidth. Additionally, achieving high isolation between MIMO elements across such a wide frequency spectrum poses a significant design challenge [34]. A combined microwave and mm-Wave 360-degree pattern diversity MIMO antenna was proposed by authors in. The designed antenna operates at microwave frequencies of 2.5 GHz, 3.5 GHz, 5.5 GHz, and 7.5 GHz as well as mm-Wave frequencies ranging 23–31 GHz. However, the antenna exhibits a lower realized gain, which may affect its overall performance and efficiency in its intended applications [35].

A dual-band four-port MIMO antenna with dual circular polarization for Wi-Fi/WiMAX applications was proposed in [36], demonstrating effective polarization diversity and isolation enhancement using compact radiator geometry. Similarly, a quad-port MIMO antenna designed for sub-6 GHz 5G NR applications was reported in [37], achieving high isolation and stable radiation performance through innovative radiator and ground plane design techniques. These works focus on the sub-6 GHz band and do not extend the operation to mm-wave frequencies, which is essential for modern 5G systems.

In contrast to previously reported dual-band and wideband MIMO antennas, the proposed design offers several distinct innovations. First, it achieves triple-wideband operation across the microwave (3–8 GHz), Ku (13.24–19.84 GHz), and mm-Wave (22–27 GHz) ranges within a single, compact 4-port MIMO structure, enabling full 5G coverage using one unified platform. Second, the antenna provides exceptionally large fractional bandwidths—90.91%, 39.9%, and 20.4% (98.3%, simulated)—without relying on multilayer substrates, complex feed networks, or external decoupling techniques typically used in literature. Third, the proposed configuration attains high isolation levels exceeding, reaching up to −33 dB for diagonal ports, without the use of additional structures. Fourth, the antenna demonstrates good MIMO performance, including ultra-low ECC (<0.065/0.0037/0.003), and good DG values (<9.7/9.99/9.985 dB), balanced MEG (−3.-4.5 dB), acceptable CCL and TARC values for a compact design. Finally, the integration of slot-based and L-shaped ground modifications enables simultaneous multi-resonant behavior and wideband enhancement while keeping the overall footprint highly compact (37.5 × 37.5 × 1.6 mm^3^). These factors collectively establish the proposed design as a significant advancement over existing 5G MIMO antennas.

The paper is organized as follows: The single patch antenna configuration is presented in Section 2. Section 3 then outlines the design process for the 4-port MIMO configuration. Section 4 offers a comparative analysis with previous work, and Section 5 summarizes the study’s significant results and insights.

## 2 Single-element antenna design

This section discusses the design configuration of the proposed multiband antenna operation at sub-6 GHz, Ku, and mm-Wave bands to facilitate the functionality of the 5G high speed communications.

### 2.1 Antenna geometry

The proposed antenna design integrates three interconnected structures on the front side, each meticulously designed to radiate efficiently within a specific frequency band. The resonance mechanism for the proposed antenna design includes:

- The triangular patch structure generates the higher-order resonances responsible for mm-wave operation.
- The rectangular section supports mid-band (Ku band) radiation.
- The monopole stub with a circular ring at the top of the structure governs the sub-6 GHz band.

**Fig 1:**
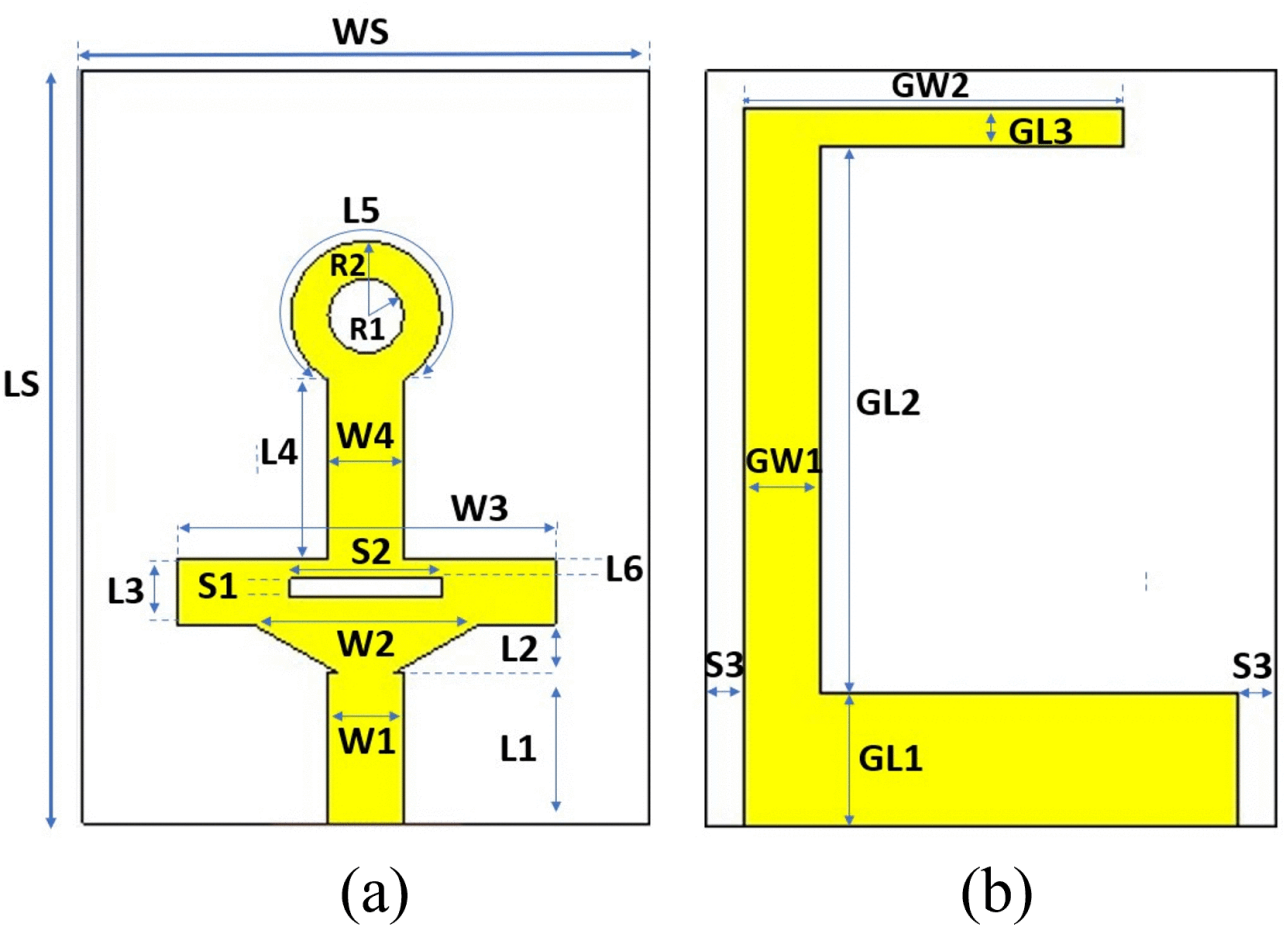
Configuration of the single–element antenna. **(a)** Front and **(b)** Back side.

Fig 1 illustrates the designed structure with detailed dimensions (a) for the front side and (b) for the back side. Where, WS = 15, LS = 20, W1 = 2, L1 = 4, W2 = 6, L2 = 1.28, W3 = 10, L3 = 1.71, W4 = 2, L4 = 4.77, L5 = 10.4, L6 = 0.5, S1 = 0.5, S2 = 4, R1 = 1, R2 = 2, GW1 = 2, GL1 = 3.5, GW2 = 10, GL2 = 14.5, GL3 = 1, and S3 = 1[all values in mm]. The single element of the MIMO antenna was designed using the CST Microwave Studio Suite software, with a 15 × 20 × 1.6 mm^3^ dimension. It utilizes the high-frequency Rogers RT-Duroid 5880 substrate material with a loss tangent (tan δ) of 0.0009 and dielectric constant (ε_r_) of 2.2.

### 2.2 Design procedure

**Fig 2:**
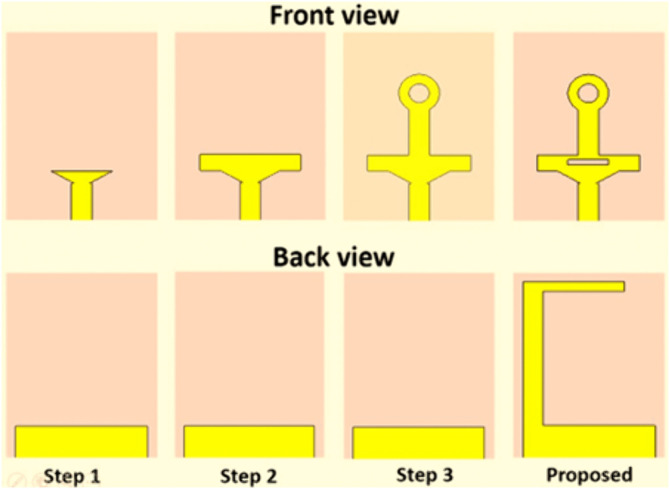
Design stages of the single antenna.


In the first stage, a 30°-30°-120° triangular patch was designed to radiate signals in a wideband frequency range within the mm-wave band. The patch was featured on top of the feedline on the front of the substrate and a partial ground plane on the rear side. The triangular patch width (W2) was calculated to radiate at 24 GHz using Equation (1) [38]. Step 1 in Fig 2 shows the designed patch in this stage.

$$\begin{array}{c} {𝐖}_{2}\approx \;\frac{2c}{f_{r}\sqrt{3{\;\epsilon }_{r}}} \end{array}$$ (1)

Where ε_r_ determines the substrate dielectric value and c is the free space light speed.

In the second stage, a rectangular radiating structure was modelled to support radiation on the Ku band frequencies. The rectangular patch width (W3) was calculated using (2) to resonate at (12 GHz) [38,39]. This structure was integrated on the top of the designed triangular patch of step 1, and the overall design shape is as illustrated in step 2 of Fig 2.


$$\begin{array}{c} W_{3}\approx \;\frac{c}{{2f}_{r}}\;\sqrt{\frac{\varepsilon_{r}+1}{2}} \end{array}$$ (2)

After integrating the structures dedicated to the millimeter-wave and Ku-band frequencies, the third stage focused on designing a structure optimized for efficient radiation within the sub-6 GHz frequency band. A single stub with a circular ring at the top was appended to the design, forming a monopole structure resonating within the sub-6 GHz range. The total stub length is defined using Equation (3) to achieve desired resonance at 6 GHz, ensuring effective radiation performance in the targeted band [35].


$$\begin{array}{c} {𝐋}_{6𝐆𝐇𝐳}={𝐋}_{4}+{𝐋}_{5}=\;\frac{\lambda_{6𝐆𝐇𝐳}}{4} \end{array}$$ (3)

Here, λ_6GHz_ is the wavelength at 6 GHz.

**Fig 3:**
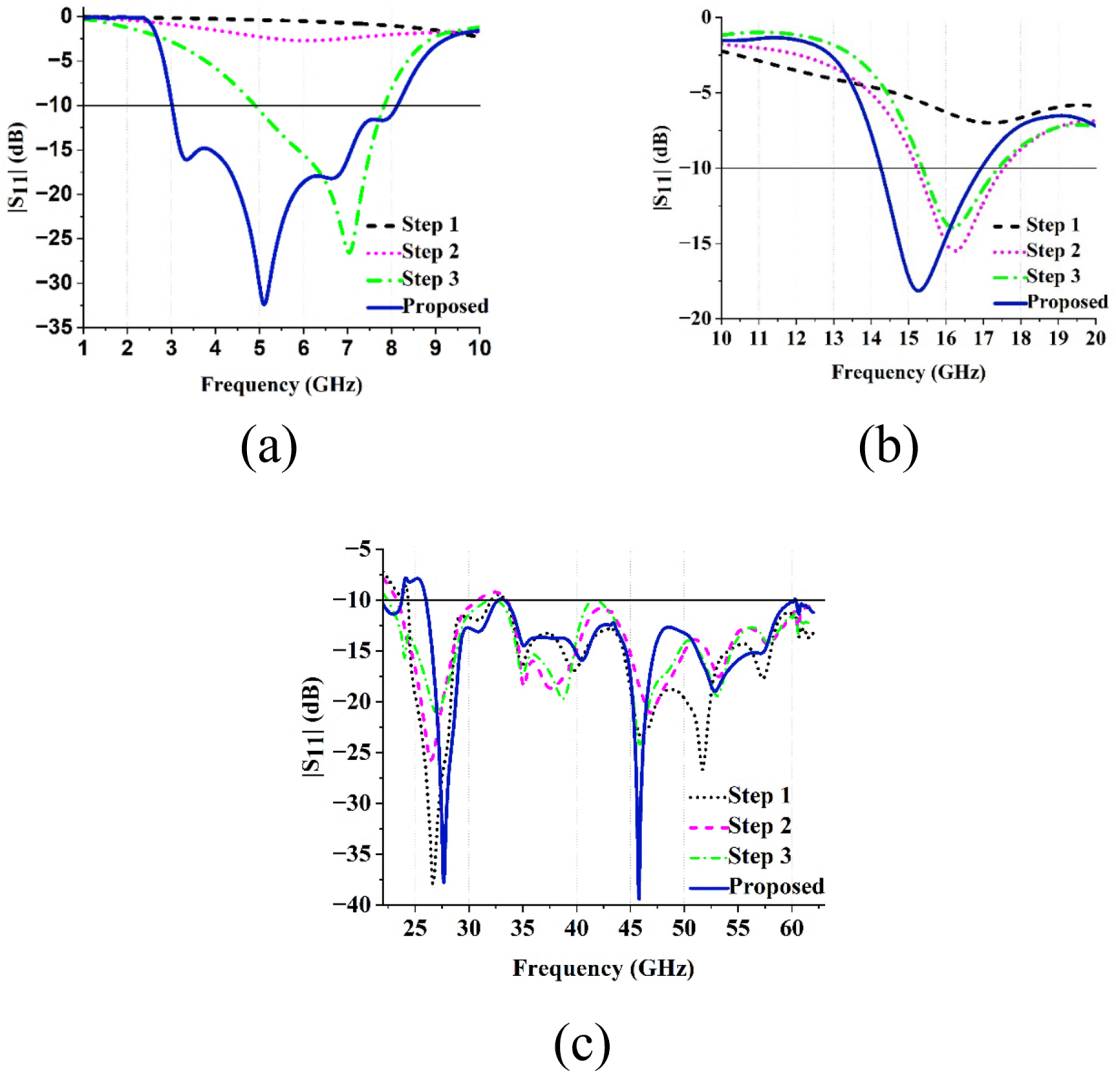
|S11| of the proposed antenna for various design stages (a) Sub-6 GHz (b) Ku and (c) mmWave bands.

However, the bandwidths of the sub 6 and ku bands are not enough, and the impedance matching of both bands is poor. To address these limitations, the final stage concentrated on optimizing the characteristics of the antenna by improving bandwidth as well as impedance matching. These enhancements were realized by introducing a slot within the rectangular section of antenna front structure and incorporating an L-shaped stub on the upper left corner of the antenna ground. The slot etched on the radiating patch modifies the current path, effectively increasing electrical length and introducing additional resonant modes. The L-shaped ground stub improves impedance matching by providing an additional current path and capacitive coupling, thereby broadening the bandwidth across all three frequency bands. The design stages of the proposed antenna and its corresponding S-parameters |S11|, are presented in Figs 2 and 3, respectively.

### 2.3 Parametric study

**Fig 4:**
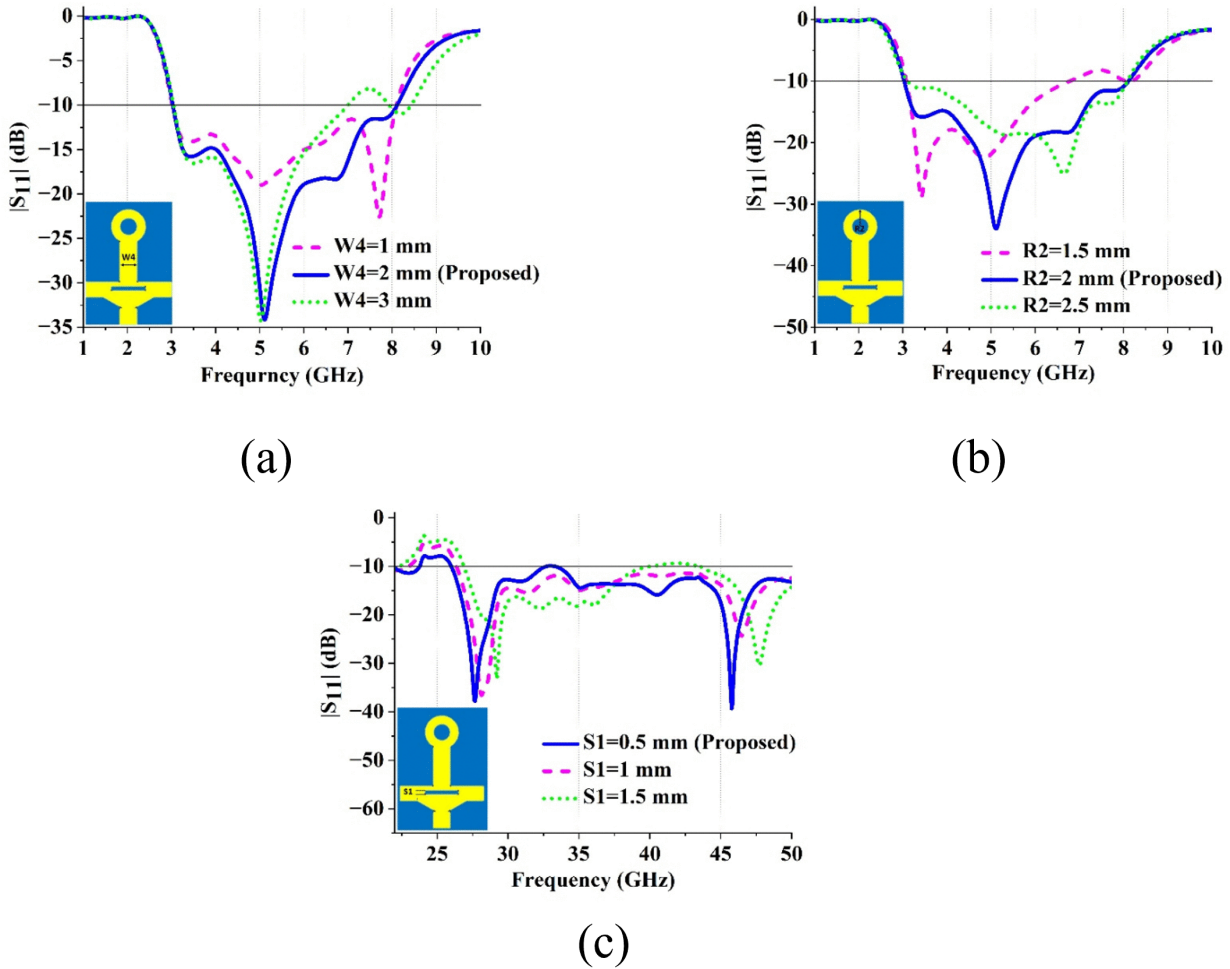
Parametric analysis of the reflection coefficients for (a) Stub width (W4), (b) Outer circle radius (R2), and (c) Slot length (S1).

Parametric studies are crucial in optimizing the proposed antenna design by analyzing the consequences of key geometric features on its performance. In this study, three parameters namely—top stub width (W4), outer radius (R2) of the ring, and slot length (S1)—were systematically tailored to evaluate their influence on the antenna reflection coefficient. The findings are illustrated in Fig 4, where (a) depicts the effect of varying the stub width (W4), (b) illustrates the impact of outer radius (R2) variation, and (c) highlights the influence of slot length (S1) variation. The above analysis highlights the significance of each parameter in achieving efficient multiband operation and meeting the design’s performance objectives. The optimal stub width (W4) was determined to be 2 mm, which resulted in a wider bandwidth. Similarly, an outer radius (R2) of 2 mm exhibited the best impedance matching with the largest bandwidth. Additionally, a slot length (S1) of 0.5 mm outperformed slot lengths of 1 mm and 2 mm in terms of impedance matching at high-frequency regions. These results emphasize that meticulous parameter optimization is critical for improving the overall performance of the proposed antenna configuration.

### 2.4 Current distribution

The investigation of the surface current distribution is fundamental to understanding the resonance characteristics of the designed antenna. Examining the surface current patterns at diverse resonance frequencies makes it possible to pinpoint the active regions of the antenna structure responsible for strong radiation. This insight validates the effectiveness of the design and serves as a guide for further optimization.

**Fig 5:**
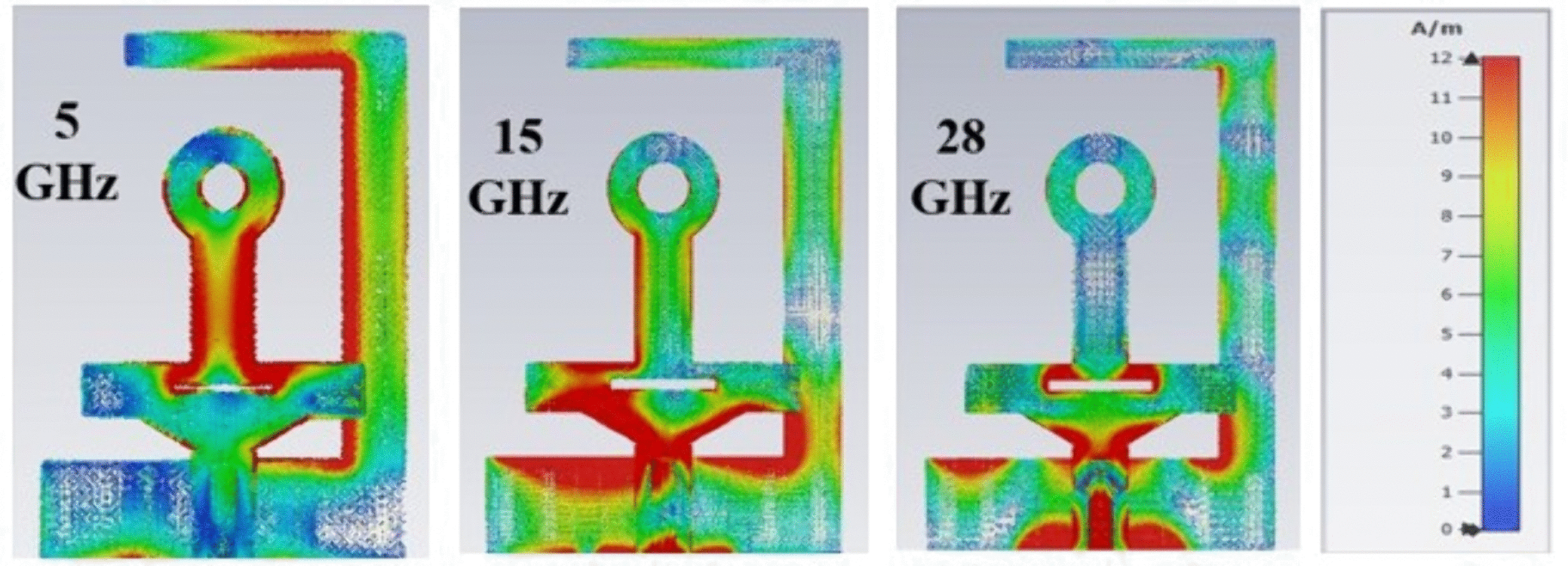
Current distributions of the single-element antenna at various resonances.

This section analyzed the current distribution to demonstrate the antenna’s capability to achieve multiband operation and efficient radiation across the targeted frequency bands. As depicted in Fig 5, the surface current distribution reveals distinct behaviors at each resonant frequency. At 5 GHz, the current density is more concentrated on both sides of the monopole stub, indicating its primary role in supporting the sub-6 GHz bands. In addition, the current density is very high along the edges of the L-shaped stub on the ground plane. This phenomenon also clarifies the significant contribution of this stub in achieving wider bandwidth. Similarly, at 15 GHz, the current gathers more at the triangular edges, at the left side of the rectangular structure patch, and the upper edges of the partial ground plane. So, this portion of the patch with the partial ground plane is responsible for the resonance in this region. Finally, at higher frequencies of 28 GHz, the triangular patch plays a critical role in establishing efficient radiation, showcasing the antenna’s suitability for mm-wave applications. This analysis underscores the effectiveness of the design in covering the intended frequency bands with high efficiency.

### 2.5 Gain and radiation efficiency

**Fig 6:**
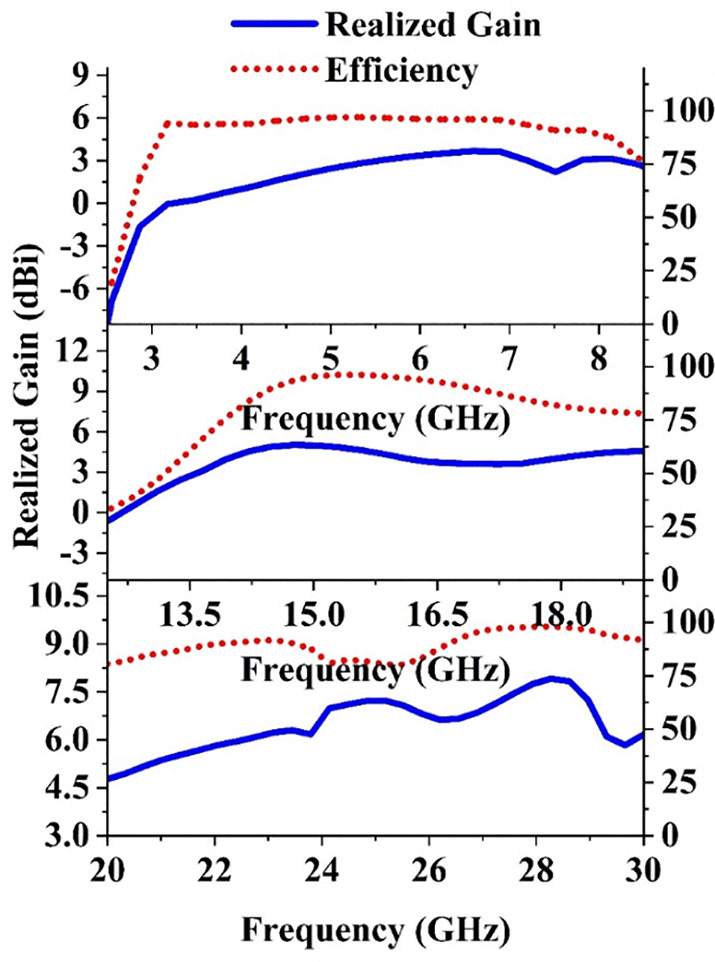
Realized gain and radiation efficiency of the proposed single antenna for all the bands.

The performance of the developed single element antenna is assessed based on its gain and radiation efficiency throughout the three operational frequency spectrums (Simulation): 3–8 GHz (sub-6 GHz), 14.2–17 GHz (Ku-band), and 26–63.9 GHz (mm-wave). The antenna’s gain was analyzed over the entire frequency range to ensure consistent performance in all bands. For the sub-6 GHz range (3–8 GHz), the antenna exhibits a peak gain of 3.7 dBi, which ensures strong signal transmission and reception for wideband communication. In the Ku band from 14.2–17 GHz, the peak gain reaches 5 dBi, which is suitable for high-frequency applications with focused beam characteristics. For the wider mm-wave band of 22–30 GHz, the peak gain achieves a maximum of 7.6 dBi. Then, the gain increases progressively with frequency, reaching its peak value of 11.3 dBi at 52.6 GHz, maintaining stable performance across this wide frequency range. The antenna’s radiation efficiency was also observed to assess the proportion of the input power radiated as useful electromagnetic waves. For the sub- 6 GHz band, the 14.2–17 GHz band and the 22–30 GHz band the efficiency achieves a peak value of about 90%, 95.6% and 96.2% respectively. Fig 6 depicts the simulated gain and efficiency plots across the frequency bands.

## 3 MIMO antenna design geometry

MIMO systems provide a technique in which multiple antennas send and receive data simultaneously. This increases data rate and improves radio transmission capacity, enabling data to travel simultaneously across many signal pathways [40]. A 4 × 4 MIMO antenna with the proposed single element was designed to confirm its applicability for high data rate applications of 5G.

**Fig 7:**
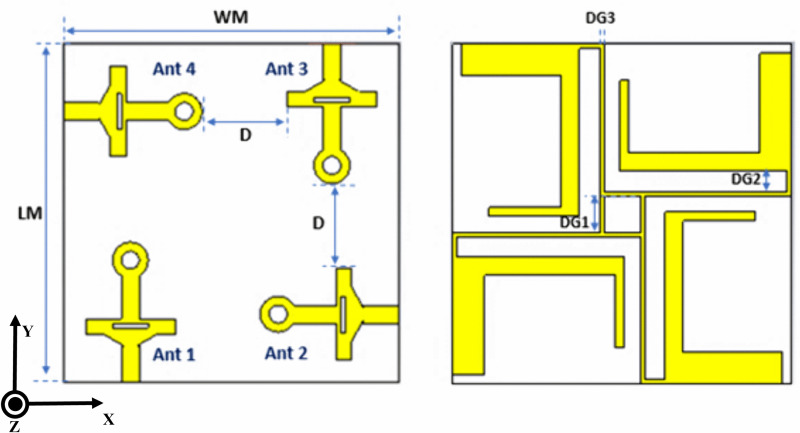
MIMO antenna configuration (a) Front and (b) Back side.

The four-port MIMO configuration incorporates single-element antennas on a compact Rogers RT-duroid 5880 substrate with space between elements equal to 9.5 mm and overall dimensions of 37.5 × 37.5 × 1.6 mm3. Fig 7 illustrates the detailed layout of the proposed MIMO antenna, where LM = 37.5, WM = 37.5, D = 9.5, DG1 = 4, DG2 = 2.25, and DG3 = 0.5 [all values in mm]. The design ensures a compact size while maintaining a good isolation characteristic.

**Fig 8:**
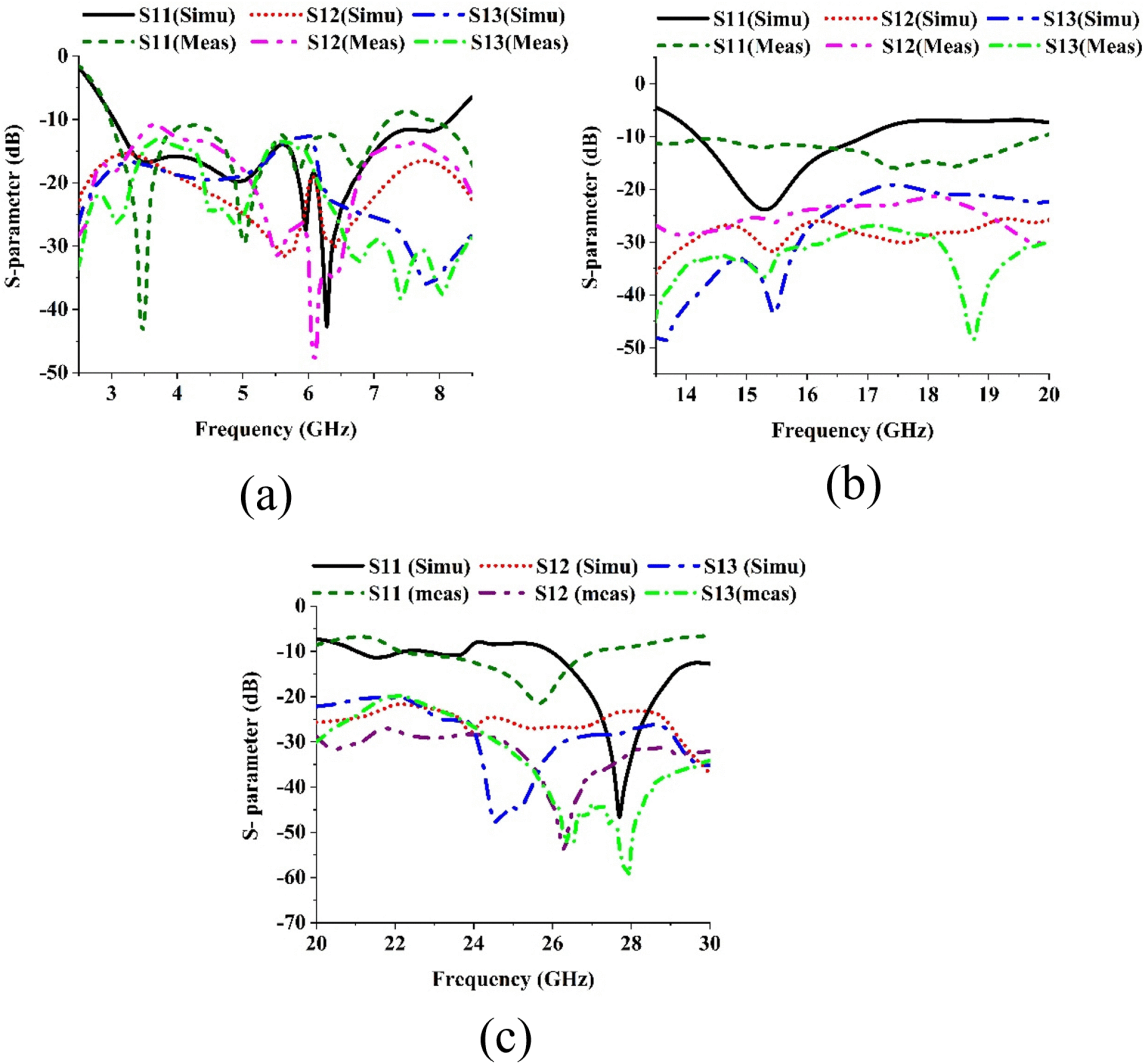
S-parameters of the suggested antenna, both measured and simulated in the (a) 5G sub-6 GHz, (b) Ku and (c) mmWave bands.

The MIMO antenna operates across three distinct frequency spectrums (sub-6 GHz, Ku, and mmWave bands). This multiband functionality addresses the diverse requirements of modern wireless communication systems, making it well-suited for advanced 5G applications. The compact size of the MIMO antenna makes it suitable for modern communication devices with limited space. The antenna elements are arranged orthogonally to act as effective decoupling mechanisms, suppressing mutual coupling between MIMO ports by generating out-of-phase surface currents. The antenna elements are arranged orthogonally to achieve high isolation between the MIMO ports. Enhanced isolation ensures reliable performance by significantly reducing interference between antenna elements, which is crucial for achieving high MIMO capacity and improving overall channel performance. The designed MIMO antenna exhibits excellent impedance matching with a return loss of less than −10 dB across all operational bands. The effectiveness of the designed multiband MIMO antenna was investigated by analyzing the S-parameters. Fig 8(a) compares the simulated and measured magnitudes of the S parameters in the sub-6 gigahertz region. When the magnitude of S11 is measured, only port 1 of Antenna 1 is excited, and the other ports are terminated by 50 Ohm terminators.

However, when the magnitudes of other S parameters like S12 and S13 are measured, two ports are excited, and the others are terminated by the 50 Ohm terminators.

The experimental data reveals that the developed MIMO prototype has a working fractional bandwidth of 90.77% ranging from 2.96–7.88 GHz for |S- parameter| < −10 dB. It is also found that at high frequencies starting from 6.04 GHz until 7.88 GHz, the mutual coupling between diagonal elements (1,3) is very low (|S13| ≤ −18 dB). The coupling |S13| is high in two regions, namely, 3.08 to 3.8 GHz and 5.32 to 6 GHz

On the other hand, the coupling between the adjacent elements (1,2) is high at the low-frequency region from 3.04 to 4.64 GHz. After that, the coupling is higher up to 7GHz. The maximum coupling within the overall bandwidth with any pair of elements (1,3) or (1,2), is not more than −10 dB. So, the overall interelement isolation performance is sufficient for the designed quad-element MIMO antenna for this band. Numerical and measured reflection coefficients were also examined for Ku and mm-Wave bands as illustrated in Fig 8(b) and 8(c). In the Ku band, the measured fractional bandwidth for |S11| < −10 dB is about 15.8%. It is also observed that the maximum mutual coupling between the adjacent elements (1,2) is only −21 dB, and the coupling is very low at the lower and upper cutoff edges of the band (−24 dB). Whereas the maximum coupling found between the angular elements is only −23 dB and here also the coupling is minimum at the two edges, especially at the upper cutoff frequency (−39 dB). For the mm wave band, the fractional bandwidth is very high but up to 30 GHz frequency the data is presented due to lack of measurement facility. Within this band the maximum coupling recorded between the adjacent elements is only −30 dB whereas the coupling between the angular elements is also very low only −33 dB.

## 4 Results and discussions

**Fig 9:**
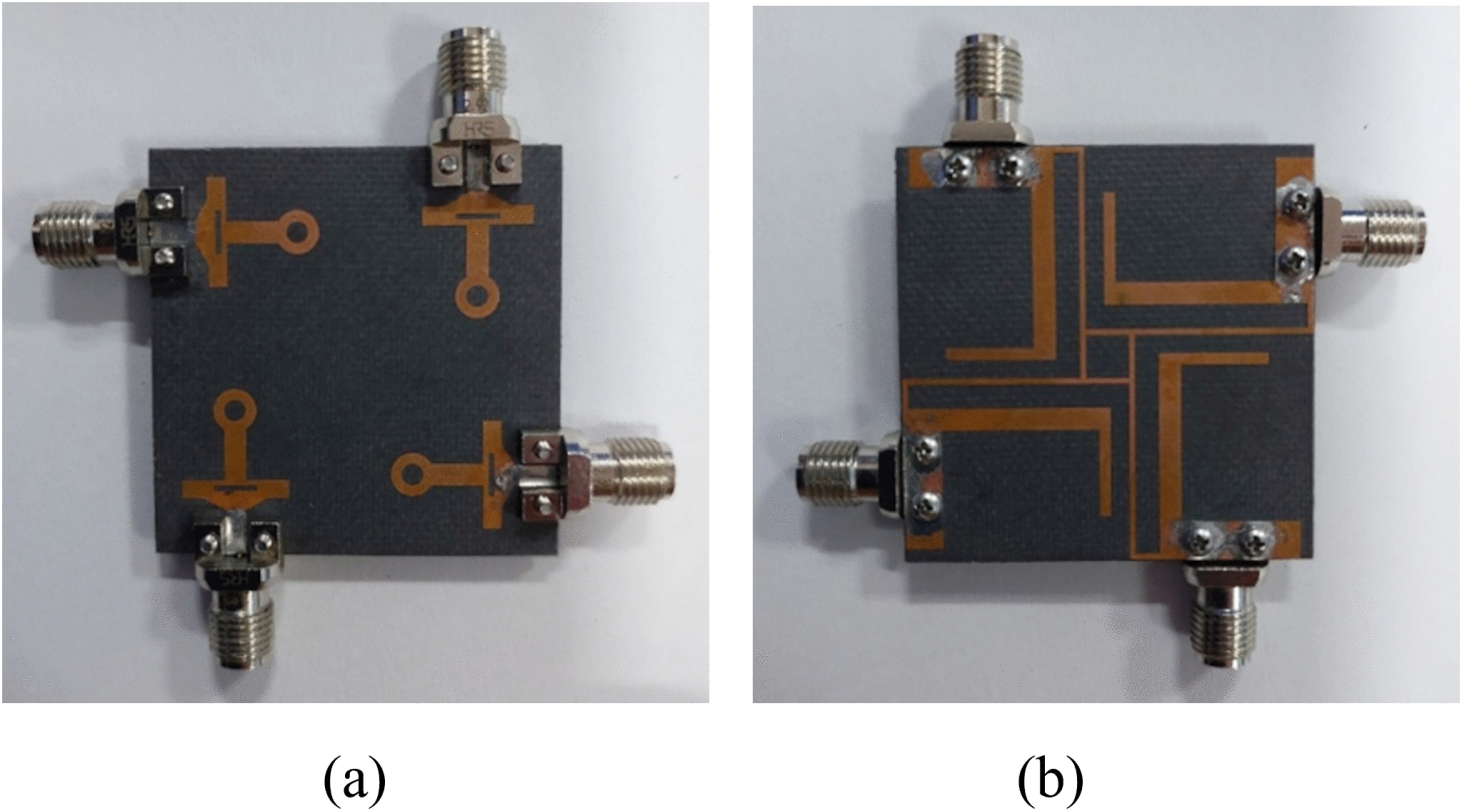
Fabricated MIMO antenna. **(a)** Front side and **(b)** Back side.

To verify the simulated performance, a 4-port MIMO antenna is fabricated using a 1.6 mm thick substrate. Fig 9 shows the front and back sides of the fabricated antenna. The prototype was measured using an Agilent Vector Network Analyzer (VNA) for the reflection and transmission coefficients and a standard anechoic chamber to evaluate gain and radiation patterns. During the measurement of each port, the remaining ports were terminated with matched 50-Ω loads to ensure accurate isolation and mutual coupling characterization. The measured |S₁₁|, isolation, and radiation characteristics exhibit close agreement with CST simulations, with minor frequency shifts attributed to fabrication tolerances and connector effects. The antenna maintains stable omnidirectional and monopole-like radiation across all three operating bands, confirming excellent measurement reliability, repeatability, and robustness of the proposed design.

**Fig 10:**
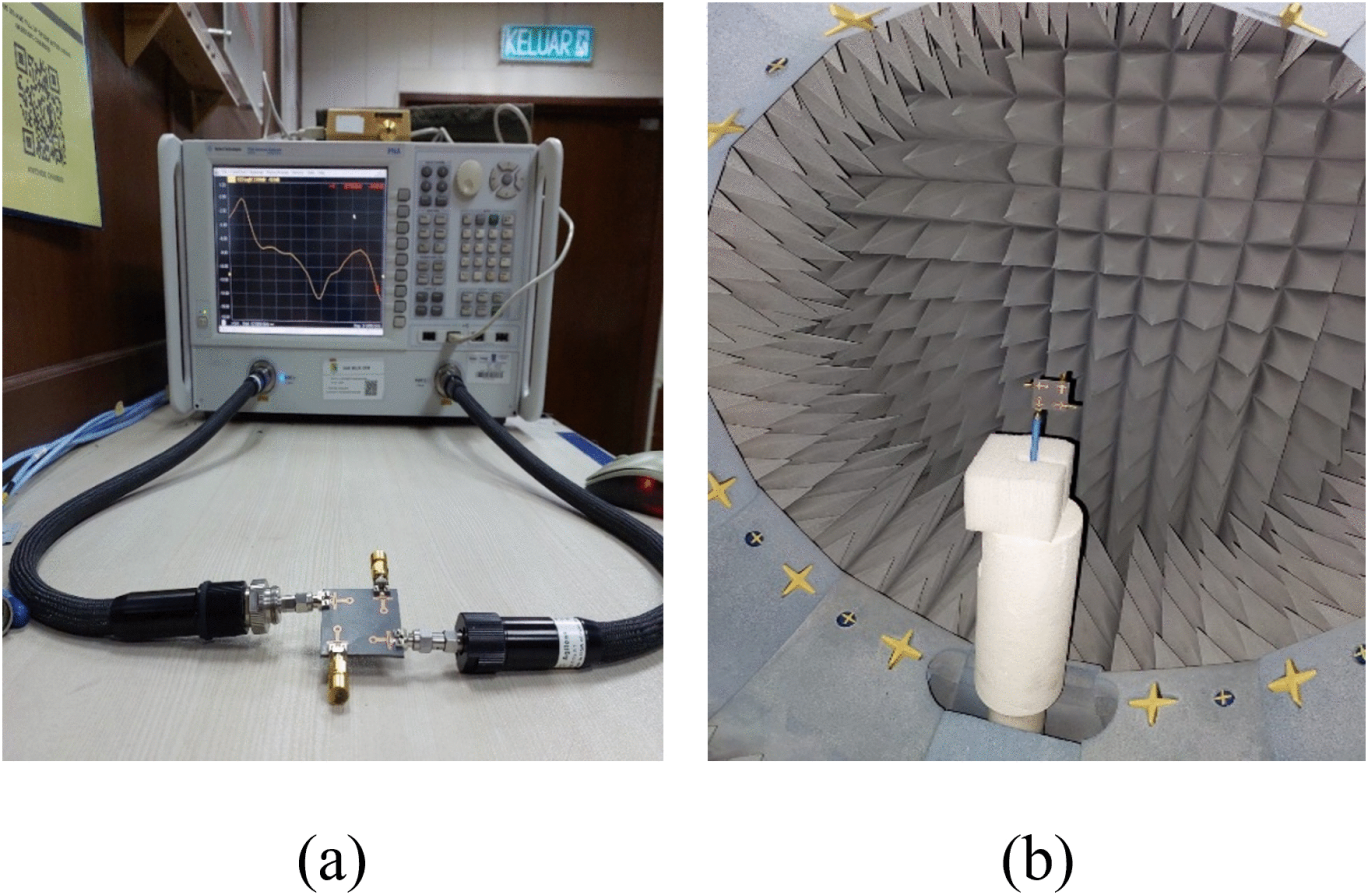
Experimental arrangement of the developed antenna (a) VNA measurement setup, (b) Satimo measurement setup.

Fig 10 shows the experimental setup to measure antenna performance with (a) VNA and (b) Satimo nearfield measurement system. The comparison between the simulated and measured S-parameters are already presented in Fig 8.

**Fig 11:**
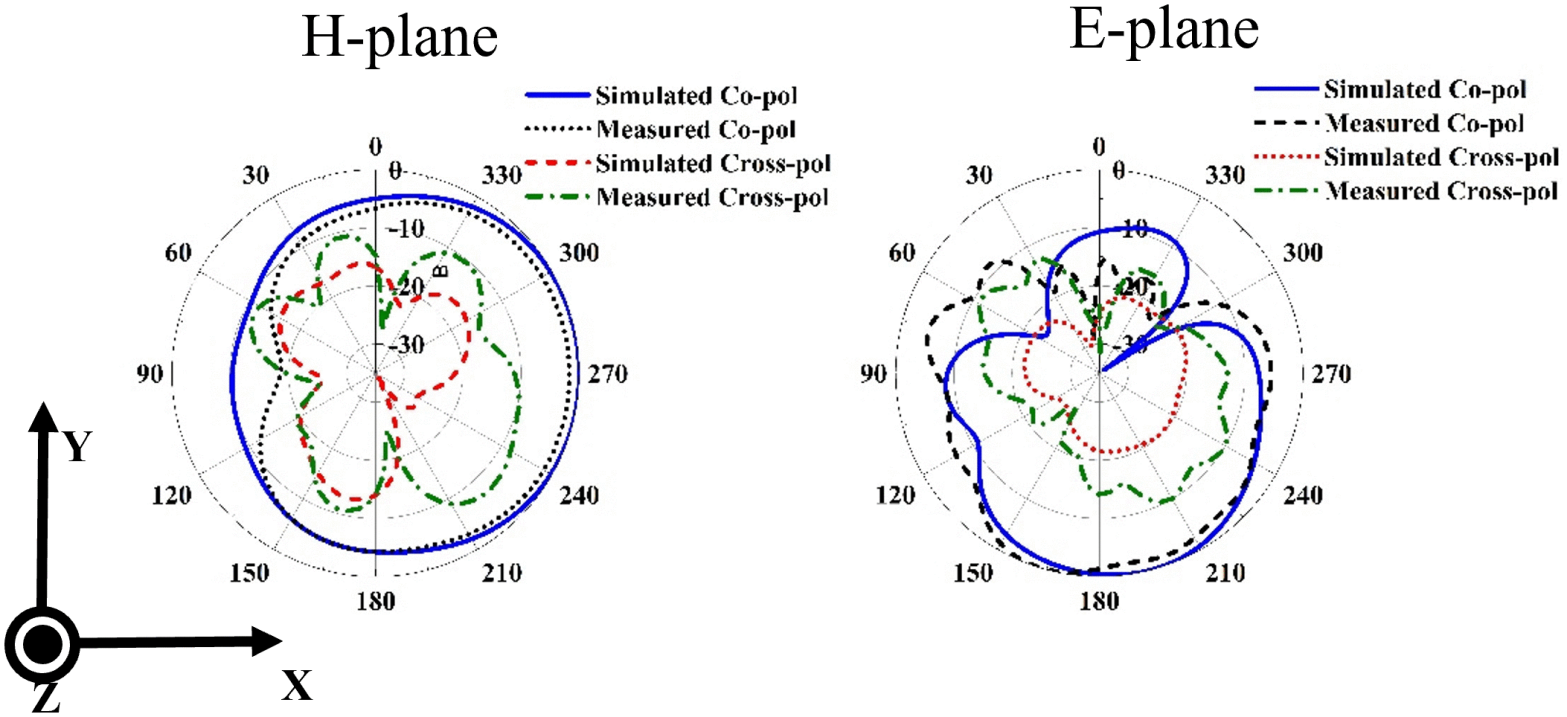
Simulated and measured radiation patterns of the proposed antenna at 6.3 GHz.

**Fig 12:**
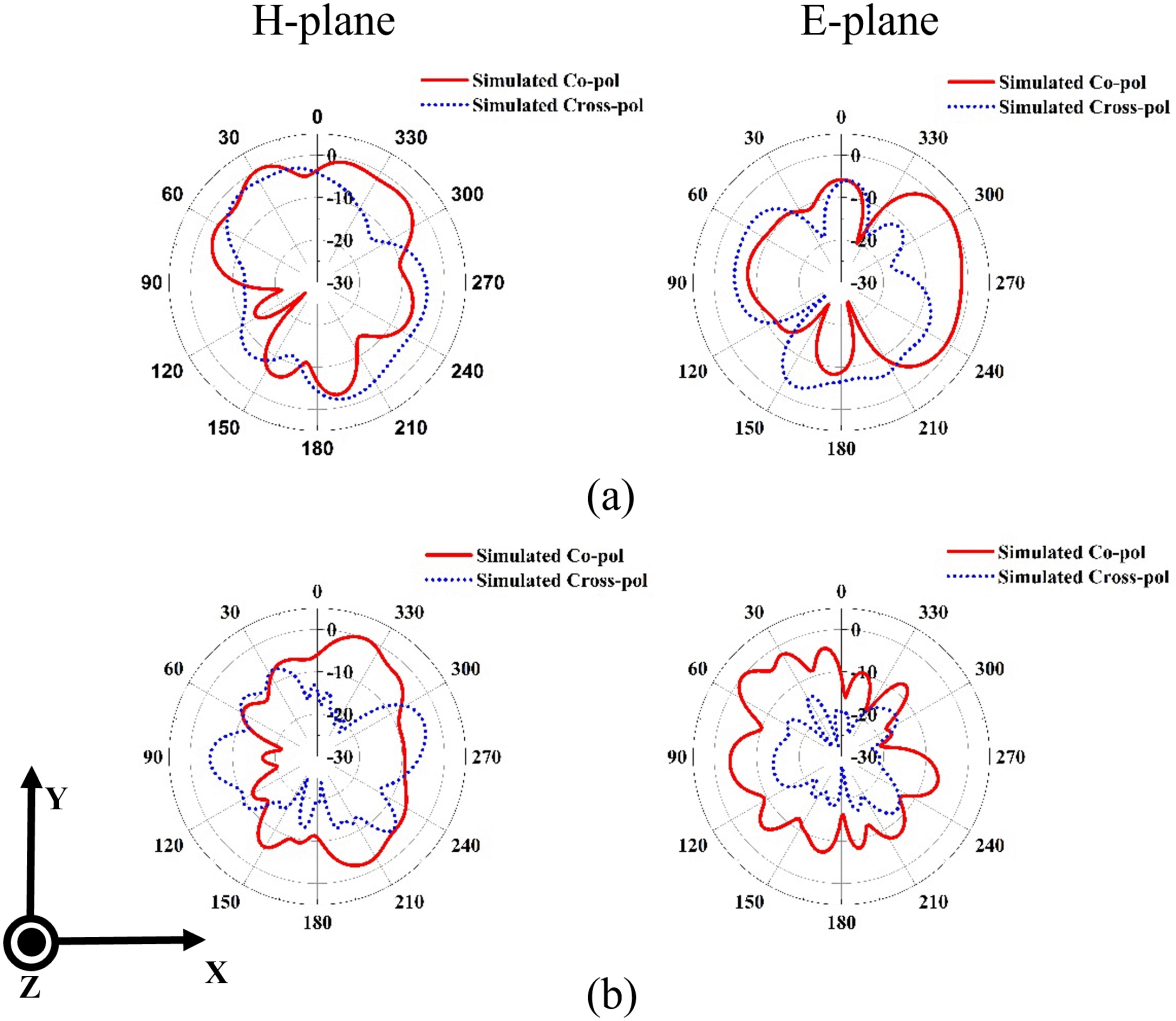
Simulated radiation patterns of the proposed antenna at (a)15.3 GHz and (b) 27.7 GHz.

**Fig 13:**
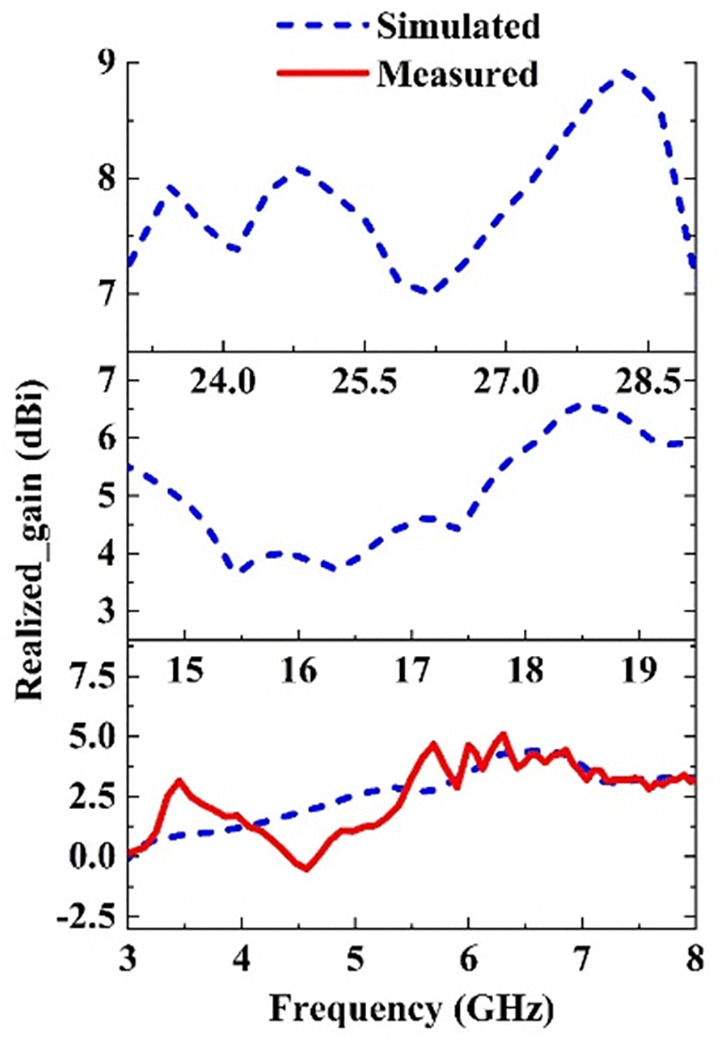
The simulated and measured realized gain of the proposed MIMO antenna.

The simulated and measured radiation patterns at 6.3 GHz in the H and E plans are given in Fig 11. The measurement results are in good agreement with the simulation. It is observed that the radiation pattern is quasi-omnidirectional in the H-plane. In the E plane, a tilted directional pattern is observed. The simulated radiation patterns at 15.3 GHz and 27.7 GHz are also presented in Fig 12. At 15.3 GHz, the H-plane radiation pattern exhibits a main-lobe direction at 27°, with a 3-dB beamwidth of 29° and the E-plane radiation pattern exhibits a main-lobe direction at 302°, with a 3-dB beamwidth of 96.7°. For the high frequency band at 27.7 GHz, the H-plane radiation pattern exhibits a main-lobe direction at 337°, with a 3-dB beamwidth of 38.8° and the E-plane radiation pattern exhibits a main-lobe direction at 114°, with a 3-dB beamwidth of 31.9°. The simulated and measured realized gain characteristics of the designed 4-element MIMO antenna across the three bands, are presented in Fig 13. At the resonant frequencies of 6.3, 15.3, and 27.7 GHz, the antenna demonstrates peak measured gains of 5, 5.5, and 8.7 dBi, respectively. Notably, the gain improves significantly as the frequency increases, attributed to the greater effective aperture of the antenna at higher frequency spectrums.

## 5 Diversity performance of the proposed MIMO configurations

The different diversity features of the proposed 4-port multiband MIMO antenna are presented and discussed by assessing the following parameters: Envelope Correlation Coefficient (ECC), Diversity Gain (DG), Total Active Reflection Coefficient (TARC), Channel Capacity Loss (CCL) and Mean Effective Gain (MEG). The specified performance metrics give a clear idea of the effects of mutual coupling on the diversity profile of the developed MIMO antenna in the targeted sub-6 GHz, Ku band and mm-Wave band frequency regions.

### 5.1 Envelope correlation coefficient (ECC)

The ECC is one of the important factors in assessing the performance of MIMO antennas; it measures the degree of coupling between successive radiating elements [34,41,42]. A low ECC value indicates weak correlation, meaning that each antenna port experiences independent fading channels. For diversity systems, independence of radiation patterns significantly improves link reliability. The ECC for ports i and j of n port MIMO can be calculated by the following equation (4) [43].


$$\begin{array}{c} {ECC}_{ij}=\frac{\sum_{k=\text{1}}^{n}S_{ik}\overset{*}{\underset{jk}{S}}}{\left(\text{1}-\sum_{k=\text{1}\;}^{n}{\left|S_{ik}\right|}^{\text{2}})(\text{1}-\sum_{k=\text{1}\;}^{n}{\left|S_{jk}\right|}^{\text{2}}\right)} \end{array}$$ (4)

Here, the transmission coefficients are denoted by S_ji_ and S_ij_, and the reflection coefficients by S_ii_ and S_jj_. Ideally, any MIMO antenna should have an ECC value of zero, however for practical applications, it is advised to maintain it around 0.5 [44]. Since the proposed four port MIMO antenna is a symmetric structure the ECC between the adjacent elements will be identical and can be approximated as:


$$\begin{array}{c} {ECC}_{adj}=\frac{\text{4}{\left|S_{\text{1}\text{1}}\overset{*}{\underset{\text{1}\text{2}}{S}}+S_{\text{1}\text{2}}\overset{*}{\underset{\text{1}\text{3}}{S}}\right|}^{\text{2}}}{{\left(\text{1}-{\left|S_{\text{1}\text{1}}\right|}^{\text{2}}-\text{2}{\left|S_{\text{1}\text{2}}\right|}^{\text{2}}-{\left|S_{\text{1}\text{3}}\right|}^{\text{2}}\right)}^{\text{2}}} \end{array}$$ (5)

Similarly, the ECC between the diagonal elements will be identical and can be approximated as:


$$\begin{array}{c} {ECC}_{diag}=\frac{\text{4}{\left|S_{\text{1}\text{1}}\overset{*}{\underset{\text{1}\text{3}}{S}}+{\left|S_{\text{1}\text{2}}\right|}^{\text{2}}\right|}^{\text{2}}}{{\left(\text{1}-{\left|S_{\text{1}\text{1}}\right|}^{\text{2}}-\text{2}{\left|S_{\text{1}\text{2}}\right|}^{\text{2}}-{\left|S_{\text{1}\text{3}}\right|}^{\text{2}}\right)}^{\text{2}}} \end{array}$$ (6)

**Fig 14:**
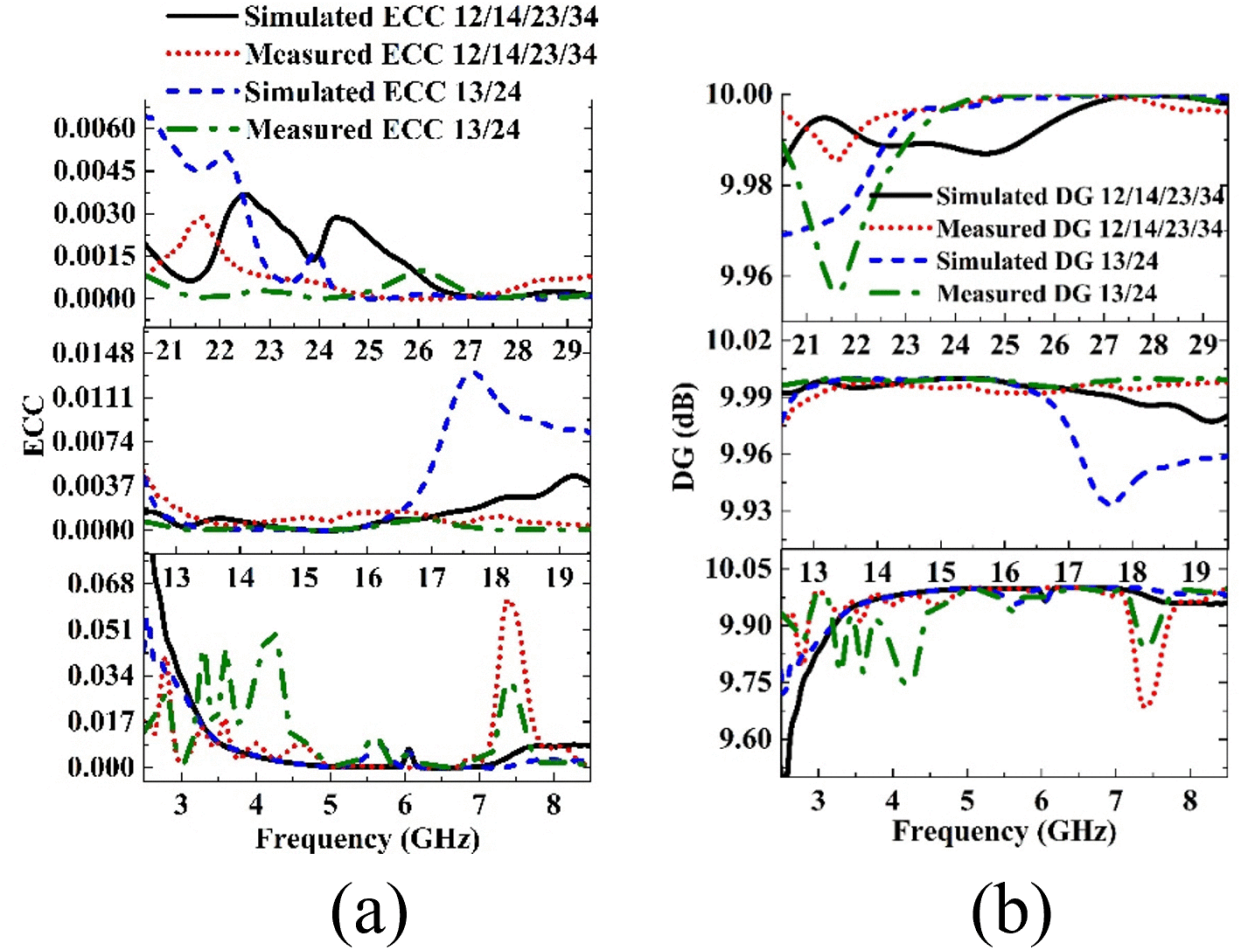
Simulated and measured (a) ECC and (b) DG values for the Sub-6, Ku and mm-Wave bands of the proposed antenna.

Using these two formulas the ECC is calculated with both simulated and measured data which is shown in Fig 14(a). It is observed from the graph that the simulated and measured ECC values between the adjacent and diagonal elements for all bands are not more than 0.07 which is far less than the threshold value.

### 5.2 Diversity gain (DG)

The DG also explains how diversity performance increases the radiated power in a MIMO antenna system. The DG is also an essential parameter used for evaluating MIMO antenna performance, representing the improvement in signal reception reliability achieved by using multiple antennas. DG is calculated related to ECC and expressed as shown in equation (7) [45,46].


$$\begin{array}{c} DG=10\;\sqrt{1-|ECC{|}^{2}} \end{array}$$ (7)

For small values of ECC (<0.2) this equation can be approximated as:


$$\begin{array}{c} DG=10\;\sqrt{1-|ECC|} \end{array}$$

where DG is in dB. The DG value of a MIMO antenna needs to be as close as possible to 10 dB. Experimental results reveal that the simulated and measured values of DG for all bands is not lower than 9.6, which is very near to 10 dB as shown in Fig 14(b).

### 5.3 Total active reflection coefficient (TARC)

**Fig 15:**
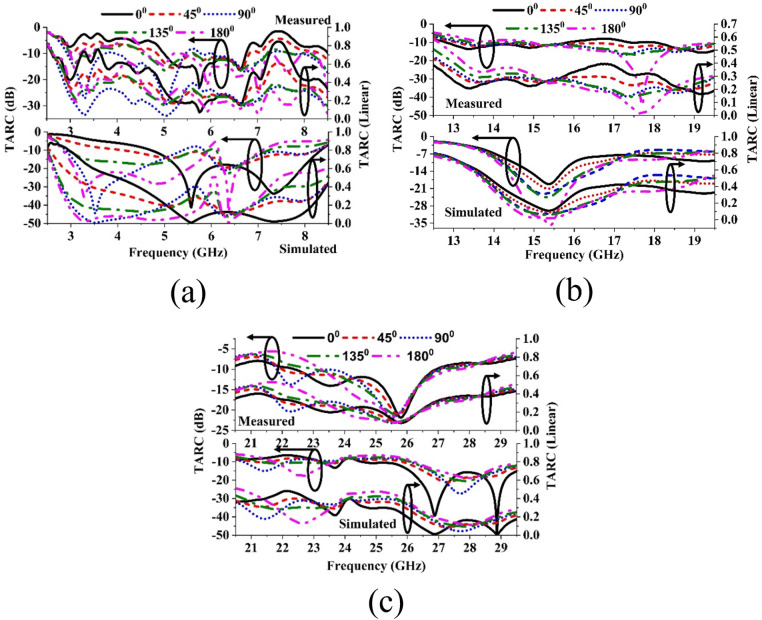
Simulated and measured results of TARC for (a) Sub 6 gigahertz band, (b) Ku band and (c) mm-Wave Bands with different phase excitation.

TARC is also an important diversity parameter to justify the efficient performance of a MIMO system. The linear value of TARC varies from 0 to 1. The closer value to 1 represents better MIMO performance. The threshold value of TARC on this scale is 0.5 whereas in dB scale this value is considered as −6 dB. Fig 15 represents the simulated and measured TARC values in both linear and dB scale for the three frequency bands with the phase excitations from 0–180 with an interval of 45.

Fig 15(a) shows that the TARC band in the sub-gigahertz frequency region which confirms that the proposed type of symmetrical structure phase 90 degree shows the best TARC scenario. At this phase both the simulated and measured TARC value remains within 0.03–0.39 in linear scale or −8.2 dB – −29.9 dB in dB scale. For the Ku band for the same phase the TARC value remains within 0.15–0.34 in linear scale or −9.37 – −16.67 in dB scale. For the mm Wave band for the same phase the TARC value remains within 0.15–0.34 in linear scale or −10.1 – −21.93 in dB scale. The level of mismatch between different phases are more for the Sub-6 GHz band but less for ku band and negligible for mm Wave band as shown in Fig 15(b) and 15(c) respectively.

### 5.4 Channel capacity loss (CCL)

The CCL quantifies the reduction in channel capacity due to correlation and mutual coupling among the antenna elements. It was computed from the measured S-parameters using the standard correlation matrix formulation. The CCL between two ports of a MIMO antenna can be determined from the following equations:


$$\begin{array}{c} CCL=\;-{𝐥𝐨𝐠}_{2}𝐝𝐞𝐭(\Psi ) \end{array}$$ (8)

$$\begin{array}{c} \Psi =\;[\begin{array}{cccc} \psi_{11} & \psi_{12} & \psi_{13} & \psi_{14}\\ \psi_{21} & \psi_{22} & \psi_{23} & \psi_{24}\\ \psi_{31} & \psi_{32} & \psi_{33} & \psi_{34}\\ \psi_{41} & \psi_{42} & \psi_{43} & \psi_{44} \end{array}] \end{array}$$

Where the diagonal terms can be defined as:


$$\begin{array}{c} \psi_{ii}\;=1-\sum_{k=1}^{4}{\left|S_{ik}\right|}^{2}=1-\left({\left|S_{i1}\right|}^{2}+{\left|S_{i2}\right|}^{2}+{\left|S_{i3}\right|}^{2}+{\left|S_{i4}\right|}^{2}\right) \end{array}$$ (9)

And the off diagonal terms can be defined as:


$$\begin{array}{c} \psi_{ij}\;=-\sum_{k=1}^{4}S_{ik}\overset{*}{\underset{jk}{S}}=-\left(S_{i1}\overset{*}{\underset{j1}{S}}+S_{i2}\overset{*}{\underset{j2}{S}}+S_{i3}\overset{*}{\underset{j3}{S}}+S_{i4}\overset{*}{\underset{j4}{S}}\right)\;(i\neq j) \end{array}$$ (10)

For the symmetrical property of the proposed design equations (9) and (10) become:


$$\begin{array}{c} \psi_{ii}=1-\left({\left|S_{11}\right|}^{2}+{2\left|S_{12}\right|}^{2}+{\left|S_{13}\right|}^{2}\right) \end{array}$$ (11)

$$\begin{array}{c} \psi_{ij}=-2Re\left(S_{11}\overset{*}{\underset{12}{S}}+S_{12}\overset{*}{\underset{13}{S}}\right)\;(off\;diagonal\;adjacent\;elements) \end{array}$$ (12)

$$\begin{array}{c} \psi_{ij}=-2\left[Re\left(S_{11}\overset{*}{\underset{13}{S}}\right)+{\left|S_{12}\right|}^{2}\right]\;(off\;diagonal\;opposite\;elements) \end{array}$$ (13)

**Fig 16:**
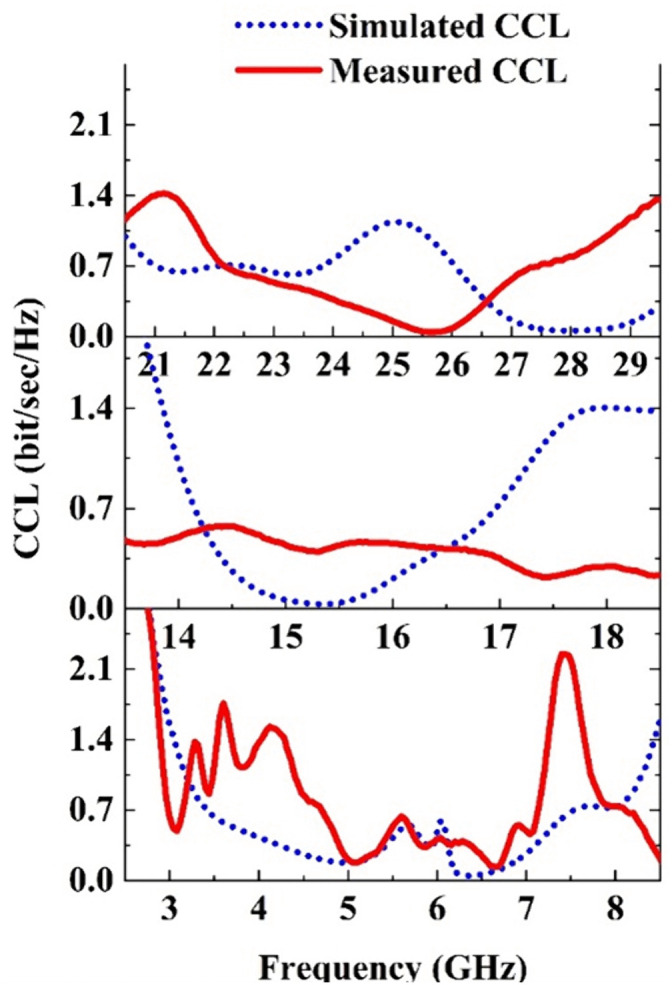
Simulated and measured CCL for the Sub-6, Ku and mm-Wave bands of the proposed antenna.

CCL value of the proposed MIMO antenna is calculated using equations (8) and (11−13). The simulated and measured CCL values are presented in Fig 16. For ku and mm Wave bands the simulated CCL value varies from 0.03–0.8 bit/sec/Hz, whereas for the Sub 6 GHz band the value varies from 0.04–1.52. In this case the high values occur at the lower and upper cut-off points and the frequency regions where either the isolation is high or the impedance matching is poor. For example, the sub-6 GHz band the isolation S13 is very high between 5 GHz − 6 GHz and for the mm-Wave band the matching is very poor between 24 GHz – 26 GHz. For this reason, the simulated CCL value is higher than other regions. More discrepancies are observed in the measured value which is due to the fabrication tolerance, more clearly the drilling and precise positioning of the signal pin on the feed line of the high frequency ports.

### 5.5 Mean effective gain (MEG)

The MEG measures the ratio of received power by each antenna element relative to an isotropic radiator in a multipath environment. MEG values were extracted using the scattering-parameter-based expression, accounting for radiation balance among the ports. The MEG of port i for a four port MIMO antenna can be calculated using the following equation:


$$\begin{array}{c} {MEG}_{i}=\frac{1}{2}\left(1-\sum_{j=1}^{4}{\left|S_{ij}\right|}^{2}\right) \end{array}$$ (14)

For the symmetric property the above equation can be simplified as below:


$$\begin{array}{c} {MEG}_{i}=\frac{1}{2}\left(1-\left({\left|S_{11}\right|}^{2}+{2\left|S_{12}\right|}^{2}+{\left|S_{13}\right|}^{2}\right)\right) \end{array}$$ (15)

**Fig 17:**
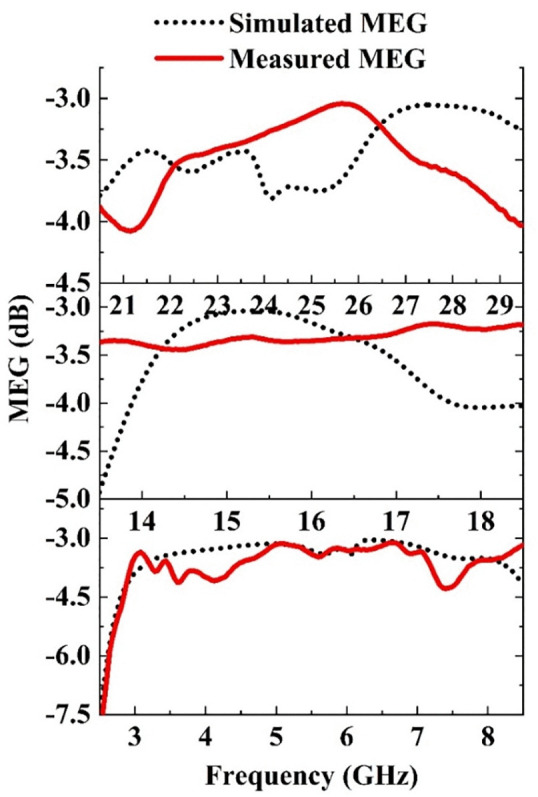
Simulated and measured MEG for the Sub-6, Ku and mm-Wave bands of the proposed antenna.

The simulated and measured MEG values are presented in Fig 17. It is found that for both the simulated and the measured MEG lies within −3 dB to −4.3 dB for all bands which lies within the acceptable limit of −3 dB to −12 dB.

## 6 Comparison

Table 1 presents a comprehensive comparison between the proposed triple-wideband 4-port MIMO antenna and recently reported multiband MIMO antennas in Refs. [29–33], considering total antenna size, operating frequency bands, impedance bandwidth, realized gain, and key MIMO performance metrics.

**Table 1:** Performance comparison of the proposed MIMO antenna with the other MIMO antenna designs.

Ref.	Total Antenna size (mm*mm*mm) (λ^3^)^a^	Frequency bands (GHz)	BW (GHz) (%)	Realized gain (dB)	MIMO Performance				
					ECC	DG (dB)	TARC (dB) (linear)	CCL (bps/Hz) (Simulated)	MEG (dB)
[29]	30 x 30 x 0.254 (0.52 x 0.52 x 0.004)	5.2–5.7 11.8–17.3 23.4–37.3	0.5 (4.6) 5.5 (37.8) 13.9 (45.8)	3.05 5.27 6.97	0.004 0.002 0.002	>9.9 >9.9 >9.9	<−10	<0.4	<−3
[30]	120 x 60 x 0.51 (0.5 x 0.35 x 0.002)	1.4–1.58 1.82–2.14 2.48–2.9 3.1–3.8 4–4.5 27.8–28.3	0.78 (2.08) 0.32 (16.16) 0.42 (15.61) 0.7 (10.14) 0.5 (11.16) 0.5 (1.78)	2.2-8.2	0.113	9.945 - 9.979	NA	NA	−4.89 − −9.31
[31]	60 × 55 × 1.2 (0.46 × 0.42 × 0.009)	2.3 - 23	20.7 (163.63)	4.5	0.002	>9.94	NA	NA	NA
[32]	80 × 80 × 0.51 (0.64 x 0.64 x 0.004)	2.38–4.13 5.04–6.12 22.28–29.28	1.75 (53.76) 1.08 (19.35) 7 (27.15)	3.47 3.5 8.5	0.284 0.112 0.021	>9.588 >9.937 >9.997	<−10	<0.4	NA
[33]	31.7 x 31.7 x 1.6 (1.057 x 1.057 x 0.053)	3-17 25.3-35.1 35.5-49.4	14 (140) 9.8 (32.45) 13.9 (32.74)	3.03 5.87 5.92	0.21 0.034 0.04	>7.86 >9.97 >9.82	<−5 <−7.4 <−6.9	<0.3	<−6
**Proposed**	37.5 x 37.5 x 1.6 (0.375 × 0.375 × 0.016)	3-8 13.24-19.84 22-27 (measured) 21-61.6 (Simulated)	5 (90.9) 6.6 (39.9) 5 (20.4) 40.6 (101.7)	5 5.5 10.1	<0.06 <0.002 <0.002	>9.68 >9.99 >9.99	<−8.2 <−9.37 <−10.1	0.14–2.25 (0.04-1.52) 0.22-0.625 (0.03-0.67) 0.04-0.8 (0.04-0.8)	−3.1 − −4.3 −3.2–3.5 −3 – −3.6

^a^λ is the free space wavelength with respect to the lowest cut-off frequency of the impedance bandwidth.

In terms of antenna size, the proposed design exhibits the most compact electrical footprint among all compared works. The physical dimensions of 37.5 × 37.5 × 1.6 mm^3^ correspond to an electrical size of 0.375λ₀ × 0.375λ₀ × 0.016λ₀ at 3 GHz, which is smaller than the electrical dimensions reported in Refs. [29–33].

Regarding operating frequency bands, Refs. [30,32], and [33] support multi band operation covering sub-6 GHz and mm-wave frequencies, whereas Ref. [29] demonstrates tri-band behavior with relatively limited bandwidth coverage. In contrast, the proposed antenna provides triple-wideband coverage across 3–8 GHz, 13.24–19.84 GHz, and 22–27 GHz (Simulated: 21–61.6 GHz), enabling unified microwave, Ku-band, and mm-wave operation within a single compact structure.

With respect to impedance bandwidth, the proposed antenna achieves −10 dB |S₁₁| measured bandwidths of 5 GHz, 6.6 GHz, and 5 GHz (Simulated: 40.6 GHz), corresponding to fractional bandwidths of 90.9%, 39.9%, and 20.4% (Simulated: 101.7%), respectively. These bandwidths are significantly wider than those reported in Refs. [29,30,32]. Although Ref. [31] demonstrates super-wideband characteristics, it does not offer more compact structure than the proposed one.

In terms of realized gain, the proposed antenna maintains stable gain values ranging from 5 to 10.1 dBi across all three bands, which are comparable to or higher than those reported in Refs. [29–33]. Notably, this gain performance is achieved without the use of external reflectors, parasitic directors, or multilayer substrates, highlighting the efficiency of the proposed planar design.

The MIMO performance metrics further distinguish the proposed antenna from existing works. The measured ECC values are extremely low (< 0.065, 0.0037, and 0.003), resulting in high DG values exceeding 9.7 dB across all operating bands. Additionally, the antenna demonstrates TARC values below −10 dB, indicating excellent active impedance matching under multi-port excitation. The CCL remains below 0.3 bits/s/Hz, and the MEG values are well balanced among all four ports, satisfying practical MIMO system requirements. In comparison, several of the referenced works either do not report all these MIMO parameters or exhibit higher correlation and reduced diversity performance.

Overall, the comparison clearly demonstrates that the proposed antenna achieves a unique combination of the smallest electrical size, ultra-wide triple-band coverage, stable realized gain, and comprehensive MIMO performance, establishing a clear advancement over recently reported multiband MIMO antenna designs for 5G wireless communication systems.

## 7 Conclusion

This work presents a compact, quad-port, ultra-wideband MIMO antenna designed for next-generation wireless communication. The proposed antenna employed two monopole structures and a stub on the front side. A partial ground plane with an L- shaped stub is used on the rear side. These modifications result in bandwidth enhancement. The MIMO configuration exhibits stable radiation patterns across all frequency bands, achieving gains of 5 dBi at 6.28 GHz and 15.2 GHz, 8.2 dBi at 27.5 GHz, with 5.1 GHz in the microwave band, 2.8 GHz in the Ku band, and 36.2 GHz in the mm Wave band. The proposed antenna covers the 5G New Radio (NR) bands n46, n47, n48, n77, n78, n79, n102 and n104 in Sub-6 GHz frequency region to support the applications for enhanced mobile broadband (eMBB), massive machine-type communications (mMTC), and ultra-reliable low-latency communication (URLLC). It also covers the 5G NR bands n257, n258, n259, n260, n261 and n262 in the mm-wave frequency spectrum to enable ultra-fast data transfer, low-latency connectivity, and high-capacity applications such as vehicle-to- everything (V2X) communication, smart cities, and industrial automation. The measured and simulated findings show strong alignment, confirming the reliability of the proposed design. The antenna also meets key diversity performance criteria, making it a promising candidate for future wireless communication systems. Future work will focus on further enhancing the performance of the gain of the proposed multiband MIMO antenna. Several techniques can be used to achieve higher realized gain without significantly increasing the antenna size such as integrating Electromagnetic Bandgap (EBG) structures, metasurfaces, and Artificial Magnetic Conductor (AMC) layers to suppress surface waves and reinforce forward radiation. Implementing these methods can further optimize the antenna’s suitability for high-data-rate 5G and emerging 6G communication systems.
